# Gene deletion of Klotho in the dentate gyrus does not affect the number of adult-born granule cells

**DOI:** 10.1038/s41598-026-54703-w

**Published:** 2026-05-27

**Authors:** Patrick Kraus, Monika Marunde, Alexandre Ryzynski, Emrah Düzel, Christoph Kaether, Michael R. Kreutz, Anja M. Oelschlegel

**Affiliations:** 1 Research Group ‘Neuroplasticity’, Leibniz Institute for Neurobiology, Magdeburg, Germany; 2 German Center for Neurodegenerative Disease (DZNE), Magdeburg, Germany; 3 Institute of Cognitive Neurology and Dementia Research (IKND), Otto-von-Guericke University Magdeburg, Magdeburg, Germany; 4 Institute of Cognitive Neuroscience, University College London, London, UK; 5 Center for Behavioural brain sciences (CBBS), Magdeburg, Germany; 6 Leibniz Institute on Aging - Fritz Lipmann Institute, Jena, Germany; 7 Leibniz Group ‘Dendritic Organelles and Synaptic Function’, Center for Molecular Neurobiology (ZMNH), University Medical Center Hamburg-Eppendorf, Hamburg, Germany

**Keywords:** Klotho, adult hippocampal neurogenesis, dentate gyrus, immature neurons, Developmental biology, Neuroscience, Stem cells

## Abstract

Overexpression and exogenous administration of the anti-ageing hormone Klotho has been shown to enhance and promote adult hippocampal neurogenesis. However, whether locally produced Klotho from dentate gyrus neurons contributes to this process has remained unclear. Here we show that conditional gene knockout of Klotho in granule cells of the mouse dentate gyrus results in a temporally restricted reduction of immature neurons (days 7–17 post-mitosis), but does not affect stem cell proliferation or long-term neuronal survival. Thus, the role of local Klotho expression is not essential for functional maturation and integration of adult-born neurons.

## Introduction

Gene deletion of Klotho results in a dramatically shortened life span due to a premature ageing syndrome^1^. During ageing, brain Klotho pools are downregulated^2,3^. This downregulation correlates with cognitive decline in both mice and humans, suggesting a link between cognitive decline in ageing and lowered Klotho brain expression^4–7^. On the other hand, higher Klotho protein levels have been shown to result in increased life expectancy both in rodents and humans^5,8^ as well as increased cognitive function in mice^5,7,9,10^.

Klotho is a transmembrane protein mainly produced by the kidney that following proteolytical cleavage is released into the blood stream. However, it has been shown that soluble Klotho does not cross the blood-brain-barrier^10–12^ raising the question whether the low level neuronal expression of Klotho has a functional role. To answer this question constitutive organismal gene knockout and overexpression studies are problematic. Not only does this approach fail to differentiate between the serum and brain pool of Klotho. This approach also results in severe secondary effects as Klotho has been linked to different mechanisms and signaling pathways in different tissues such as vessels, muscle and kidney to name a few^13^. In addition, it is difficult to assess whether the observed cognitive phenotypes are secondary effects of the premature ageing syndrome due to systemic Klotho depletion from the whole organism.

Klotho has been reported to be expressed throughout the brain in neurons and more prominently in granule cells of the dentate gyrus (DG)^14,15^. One proposed mechanism by which Klotho might affect cognitive function is through the upregulation of adult hippocampal neurogenesis (AHN)^7,15–17^, a process critical for DG dependent memory function. However, different Klotho pools may have distinct effects on AHN and whether endogenous DG Klotho specifically contributes to this process has remained unclear due to the absence of suitable conditional mouse lines. AHN is central for spatial memory formation^18–23^ with age-dependent downregulation contributing to cognitive decline^24–29^. Investigation of the regulatory mechanisms with the aim of increasing Klotho levels in the brain could lead to important therapeutic interventions for preserving cognitive function during ageing.

## Methods

### Mouse housing and ethics

Mice were bred in the core animal facility of the Leibniz Institute for Neurobiology. They were housed in standard IVC-cages in a 12-hour light/dark cycle with *ad libitum* access to food and water and supplemented by enrichment. Cages were changed regularly, but kept the same for the time of BrdU injections. Experiments were conducted with mice that were either 3 or 12 months old at the start of the BrdU injections and were sacrificed between 1 and 28 dpi. All experimental groups, across genotypes, ages, and sacrifice time points, included both male and female mice. Body weight ranged in young mice from 21.0 g +/- 0.3 g in females to 26.8 g +/-0.8 g in males and in older animals from 34.5 g +/- 0.8 g in females to 39.8 g +/- 1.1 in males (BrdU injection D1). All animal experiments were performed according to the Directive of the European Communities Parliament and Council on the protection of animals used for scientific purposes (2010/63/EU) and were approved by the competent authority at the federal state level of Saxony-Anhalt, Germany (Landesverwaltungsamt für Verbraucherschutz und Veterinärangelegenheiten, Referat 203; English: State Office for Consumer Protection and Veterinary Affairs, Department 203) under license number 42502-2-1598 LIN. Animal experiments requiring approval (§ 7 VersTierV, German Animal Welfare Laboratory Animal Ordinance) were conducted in compliance with these regulations.

### Generation of Klotho DG specific knockout mice

All experiments were performed with B6;Cg-Kl< tm1.1Arte > Gt(ROSA)26Sor< tm1(EYFP) Cos > Tg(Pomc-cre)1Lowl/Linmd mice, in which Klotho can be deleted specifically by POMC-Cre recombinase (^30^; B6.FVB-Tg(Pomc-cre)1Lowl/J, JAX stock #010714) from the granular cells of the DG (KO; Klotho-flox lox/lox; Pomc-Cre positive; Rosa26-lsl-YFP tg/tg). A YFP reporter signal is activated through Cre-mediated excision of a stop-sequence located within the lox/lox flanking site at the end of the Klotho gene and before the inserted YFP-tag. This results in YFP expression in tissue when the Klotho gene is deleted by Cre recombinase expression, thus showing Cre activity in the target area. Klotho expressing, Cre recombinase negative littermates were used as controls (WT; Klotho-flox lox/lox; Pomc-Cre negative; Rosa26-lsl-YFP tg/tg). To generate the experimental animals, original Klotho-flox (lox/lox) and YFP (tg/tg) carrying females that were kept as separate mouse line (B6;Cg-Kl< tm1.1Arte > Gt(ROSA)26Sor< tm1(EYFP)Cos>/Ckae)^31^, were bred with DG-Klotho-deficient male mice.

### Fluorescence in situ hybridization (FISH)

Twenty-µm-thick fresh-frozen slices of perfused or unperfused mouse brains were mounted on Superfrost+ slides (Epredia) and treated according to the manufacturer’s protocol (RNAscope^®^ Multiplex V2 kit). Briefly, after fixation with ice cold PFA (Roth) and ethanol (Fisher Chemical) dehydration, RNA molecules were targeted with a probe specific for Klotho (Probe Mm-Kl-C3, Catalog number 422081-C3) which was then sequentially bound by amplifying molecules and fluorophores, allowing visual detection of single mRNA molecules.

### FISH imaging and analysis

Images were acquired using a Leica SP8 STED 3X microscope with z-stacks collected at 2-µm intervals. Images were deconvoluted using the Hygens (v 24.10.0) software. Afterwards a maximum projection was generated with Fiji (ImageJ 2.9.0) and analyzed with QuPath^32^. Statistical differences between groups on nested data were analyzed with linear mixed-effect models. Parameter estimation was done using the *lmer* function implemented in the *lme4* R package. To test the significance of condition (genotype) in the model, a likelihood ratio test against a reduced model was performed in which condition was removed (*lmerTest* R package). The full model included the following parameters: Measured parameters ~ condition + (1 | animal).

### BrdU in vivo injections

Bromdeoxyuridine (BrdU, Sigma Aldrich #B5002; 10 mg BrdU/ml, in 0.9% NaCl, Fisher Chemical; dissolved via sonication) was injected intraperitoneally (i.p.; 50 mg/kg body weight) nine times, each injection 8 h apart, thus allowing comprehensive labeling of newly synthesized cells that incorporate BrdU during cell division.

### Euthanasia

Brains were collected post mortem for further analyses. Animals were euthanized either by CO_2_ inhalation followed by decapitation or died during perfusion.

For CO₂ euthanasia, animals remained in their home cage (Tecniplast GM500 IVC; approximately 5.2 L free air volume after bedding, food, and water) and were continuously monitored throughout the procedure. CO₂ was introduced at an initial flow rate of 1.435 L/min, corresponding to a volume displacement rate of approximately 28% of the cage air volume per minute. Animals were observed until they became drowsy, at which point the CO₂ flow was gradually increased in a stepwise manner until respiration ceased. Gas flow was maintained for an additional minute following respiratory arrest to ensure complete euthanasia. Animals were then immediately decapitated, brains were removed, snap-frozen in liquid nitrogen, and stored at − 80 °C.

For perfusions, mice were injected intraperitoneally with an overdose of 0.2 ml Ketamine/Xylazine (7.5 mg Ketamine, 1.5 mg Xylazine in 0.9% NaCl; Bela-Pharm, WDT, Fisher Chemical) and transcardially perfused with NaCl (0.9%), followed by PFA (4%, Roth) using an infusion pump (Cyclo I, Roth). Brains were isolated and fixated in PFA at 4 °C overnight, afterwards dehydrated for 24 h in 0.5 M sucrose (Fisher Chemical) solution and for further 24 h in 1 M sucrose solution. Then they were snap-frozen with liquid nitrogen and long-term stored at -80 °C.

### BrdU and YFP Immunohistochemistry (IHC)

For immunohistochemistry frozen brains were cut into 40-µm-thick slices using a Cryostat (CM3050, Leica). Slices were then washed 3 times in PBS (Roth) and incubated with HCl (2 M; Roth) for 60 min at room temperature. This antigen retrieval step results in DNA denaturation allowing the injected and into DNA incorporated BrdU to be accessible for the anti-BrdU antibodies. Afterwards, slices were neutralized by washing 3 times in Na-Borate Buffer (0.1 M, pH = 8.6; Sigma-Aldrich), washed again 3 times with PBS and then incubated with blocking buffer (PBS, 0.5% fetal bovine serum, 0.3% Triton-X-100; Roth, Capricorn, AppliChem) for 1 h. Following this, slices were incubated with a combination of antibodies (rat anti-BrdU abcam #ab6326, guinea-pig anti-GFAP SySy #173004, rabbit anti-SOX2 abcam #ab97959, rabbit anti-DCX abcam #ab18723, rabbit anti-NeuN abcam #177487, rabbit anti-S100B abcam #ab52642) dissolved in blocking buffer (1:500) at 4 °C in a dark chamber on a shaker for 48 h. Three washes with PBS were followed by 24 h of incubation with secondary antibodies (Alexa Fluor goat anti rat 488, Alexa Fluor donkey anti rabbit 568, Alexa Fluor goat anti guinea pig 647; Invitrogen) dissolved in PBS at 4 °C in a dark chamber. Slices were washed again 3 times with PBS and counterstained with DAPI (1:1000 in PBS; Sigma-Aldrich) for 10 min, washed 3 times with PBS and mounted on slides with coverslips using anti-fade medium (Mowiol 488 Merck Milipore).

For YFP-Immunohistochemistry 40-µm-thick slices of the same animals were stained with DAPI and 3 times washed with PBS before being mounted. YFP-signal was not observable in the BrdU-IHC as acid-sensitive YFP is deteriorated by the HCl-DNA-denaturation step.

For DCX-staining a slightly modified protocol was used due to acid-sensitivity of the DCX-staining: 40-μm-thick slices were washed 3 times in PBS, then incubated with blocking solution, before DCX antibody was incubated for 24 h. Three washes in PBS were followed by secondary antibody incubation (Alexa 568 goat anti rabbit, Invitrogen; 1:500 diluted in PBS) for 24 h. Post-fixation was done with Zamboni solution („Lana’s fixative“, Morphisto #22773) for 24 h at 4 °C in a dark room, which results in DCX antigen preservation^33^. Afterwards slices were washed 3 times with PBS, then HCl (2 M) was applied for 1 h at room temperature, followed by the above described BrdU IHC protocol.

### IHC microscopy and analysis

Images were obtained at 20x magnification using a Leica SP8 confocal microscope. For each slice, a tile scan of the whole DG in both hemispheres was acquired as a z-stack at 4-µm intervals. Images were analyzed using Fiji (ImageJ 2.9.0) in the following way: Images were adjusted for brightness/color. A maximum projection of the DAPI staining was used to calculate the respective DG volume by measuring the area of the supra- and infrapyramidal blade as well as the hilus and multiplying this with the thickness of the slice. Cells were counted using the ImageJ Cell Counter plugin by manually scoring every BrdU-positive (BrdU^+^) cell while scrolling through the z-stack. Cells were classified according to their position within the subgranular zone, defined as a one-cell-wide layer between the hilus and the granule cell layer. BrdU^+^ cell density was extrapolated by normalizing the total number of BrdU^+^ cells to the DG volume. The mean BrdU^+^ cell number for each animal was calculated by analyzing at least four images per mouse (both hemispheres). For co-stainings, a total of four images per mouse were analyzed in the same way as BrdU-stained images. Images were analyzed blind and statistics were performed with GraphPad Prism (v10.6.1).

### Statistical analysis

Statistical analyses were performed using GraphPad Prism (v10.6.1; GraphPad Software, USA) and R (lme4 and lmerTest packages for linear mixed-effect models on nested data). Type I error was set at α = 0.05. Details of the specific statistical tests used for each experiment are described in the respective figure legends and Methods subsections. Error bars indicate s.e.m. and significance levels are indicated as * *p* < 0.05, ** *p* < 0.01, *** *p* < 0.001, **** *p* < 0.0001; ns, not significant.

## Results

### DG Klotho KO results in transient changes in adult hippocampal neurogenesis

**Fig. 1:**
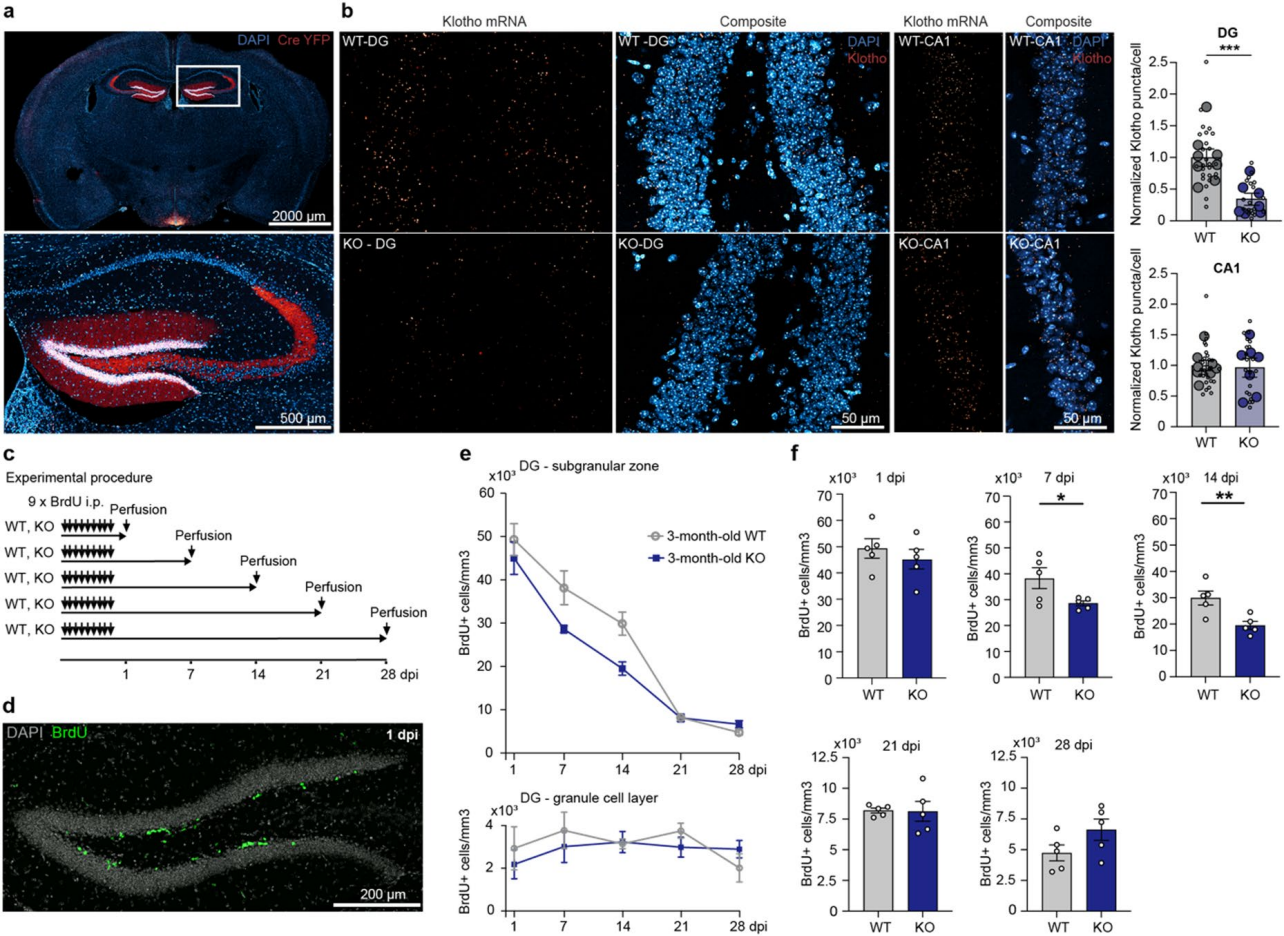
Conditional Klotho knockout in the DG results in transient reduction of immature adult-born neurons. (**a**) Coronal section of brain (upper image) and hippocampus (lower image) with POMC-Cre YFP reporter expression from 3-month-old Cre+ Klotho knockout mouse (BREGMA AP -1.6 mm). (**b**) Representative images of Klotho mRNA expression in the DG and CA1 of Klotho WT and KO mice and quantification of Klotho mRNA transcripts in the DG (*p* = 0.0007) and CA1 region. Animals = large circles (*n* = 8 WT, 7 KO), slices = small circles (DG: 26 WT, 20 KO; CA1: 27 WT, 20 KO). (**c**) Experimental design for BrdU injections. Animals were sacrificed at 1, 7, 14, 21 and 28 dpi (days post injection, after last BrdU injection) respectively to investigate different stages of AHN, with 5 KO and 5 WT sacrificed at each time point. (**d**) Representative image (20x magnification) of BrdU IHC in 3-month-old Klotho mouse at 1 dpi. (**e**) Time course evaluation of BrdU^+^ cells in the SGZ and the GCL of the DG in 3-month-old Klotho WT and KO animals. (**f**) BrdU^+^ cells in the SGZ at 1, 7 (*p* = 0.0492), 14 (*p* = 0.0097), 21, 28 dpi in 3-month-old Klotho WT and KO animals, *n* = 5 WT, 5 KO. For statistical analysis either linear mixed-effect model (b) or two-tailed students t-test were performed (f), with error bars indicating s.e.m. and * *p* < 0.05, ** *p* < 0.01, *** *p* < 0.001. Images (a, b, d) were obtained using a Leica SP8 confocal microscope and analyzed using Fiji (ImageJ 2.9.0).

To assess the role of Klotho in granule cells for adult hippocampal neurogenesis we generated DG-specific Klotho-deficient mice. POMC-Cre yellow fluorescent protein (YFP) reporter expression shows specific Cre recombinase expression limited to the DG and few POMC expressing hypothalamic neurons (Fig. 1a). Gene knockout was confirmed by immunohistochemical detection of Cre-expression (Fig. 1a) and fluorescence in situ hybridization (Fig. 1b) showing a significant reduction of Klotho mRNA transcripts selectively in the DG with no change in other hippocampal subfields such as cornu ammonis 1 (CA1).

To assess whether Klotho-deficient mice show impairment in AHN, mice were injected with BrdU and the number of newly synthesized BrdU-positive (+) cells in the DG was quantified. As AHN consists of several stages, multiple groups of mice were sacrificed at different time points (Fig. 1c) and BrdU^+^ cell numbers were quantified via immunohistochemistry (Fig. 1d). Initially AHN results in the production of a relatively high number of neuronal progenitors in the DG subgranular zone (SGZ) with only a small fraction surviving, maturing and integrating into the network (Fig. 1e). Tracking the survival of adult-born neurons revealed a significant reduction in BrdU⁺ cells in Klotho-deficient mice compared with controls at 7 dpi, when the labeled neurons were approximately 7–10 days old. A similar difference was observed at 14 dpi, when neurons were around 14–17 days post-mitosis. At later stages (21–28 dpi) no difference in cell numbers was observed compared to controls (Fig. 1f). Collectively, the data show a deficit in AHN restricted to the survival of immature postmitotic neurons between day 7 and 17. Most importantly, when we analyzed the number of BrdU^+^ cells in the granule cell layer (GCL) as a surrogate for neuronal migration to deeper layers of the DG, no difference was detected (Fig. 1e). Thus, loss-of-function of Klotho reduces survival of adult-born neurons at early stages without affecting later phases of AHN, i.e. long-term survival and migration.

### DG-specific gene deletion of Klotho does not affect stem cell pools, neuronal progenitor cell proliferation or gliogenesis

**Fig. 2:**
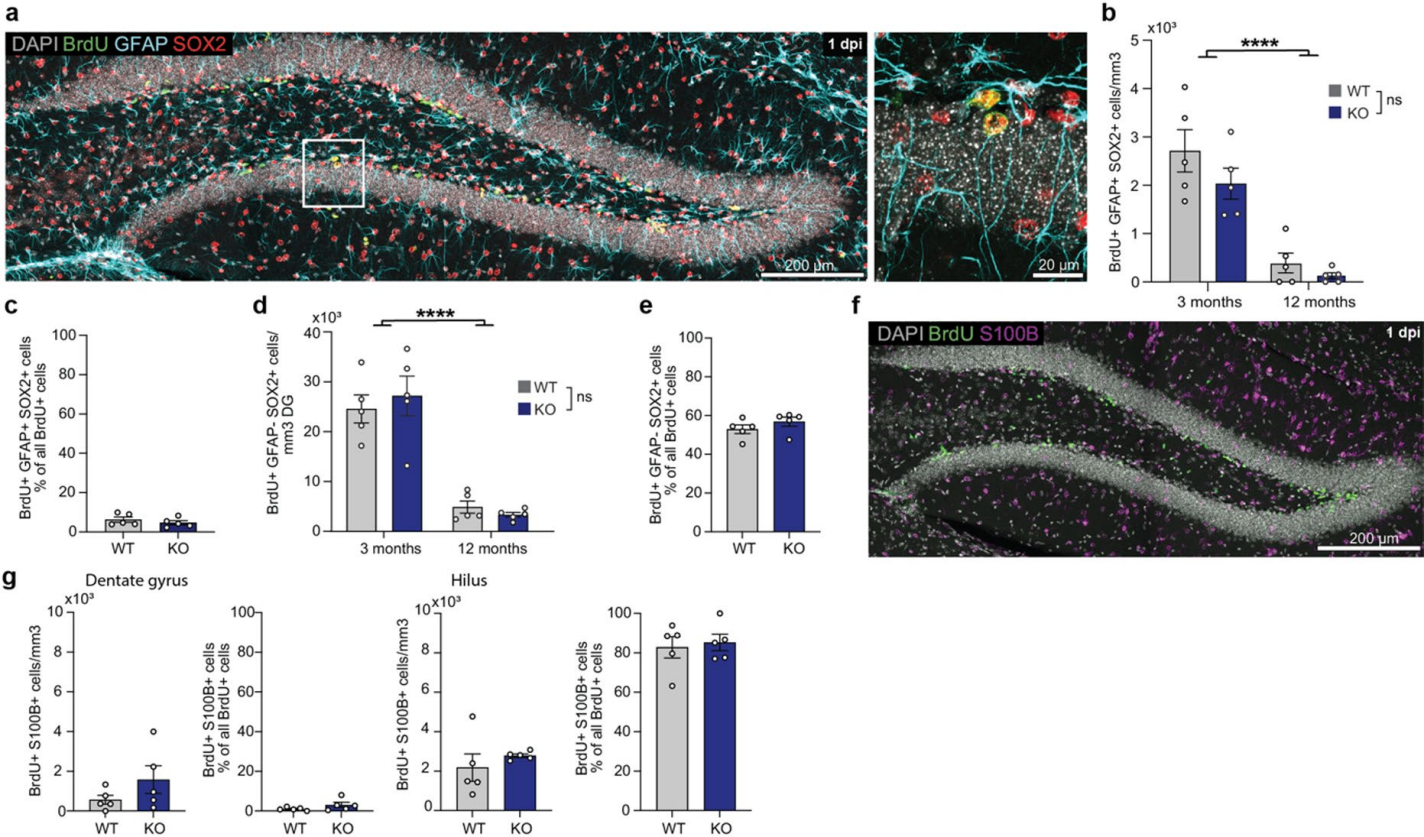
DG-specific Klotho does not alter stem cell pools, neuronal progenitor cell proliferation or adult gliogenesis. (**a**) Coronal section of the DG showing staining with neurogenesis markers BrdU, GFAP (Glial fibrillary acidic protein), SOX2 (SRY-box transcription factor 2) with one respective stem cell and TAP shown in magnification on the right. (**b**) Quantification of stem cells (BrdU^+^, GFAP^+^, SOX2^+^) in young and aged mice shows a strong age effect (*p* < 0.0001). (**c**) Relative number of stem cells at 1 dpi in young mice. (**d**) Quantification of TAP cell numbers (BrdU^+^, GFAP^-^, SOX^+^) in young and aged animals shows an age effect (*p* < 0.0001). (**e**) Relative number of TAPs at 1 dpi in young mice. (**f**) Coronal section of the DG showing the staining of adult-born glia (BrdU^+^, S100B^+^) in 3-month-old animals. (**g**) Quantification of absolute and relative numbers of adult-born glia in the SGZ and the larger adult-born glia population in the hilus in young mice. For all graphs *n* = 5 WT, 5 KO and error bars indicate s.e.m. Either two-tailed Students t-test (c, e, g) or two-way ANOVA (b, d) were performed with **** *p* < 0.0001. Images (a, f) were obtained using a Leica SP8 confocal microscope and analyzed using Fiji (ImageJ 2.9.0).

With additional immunohistochemical markers such as GFAP and SOX2 (Fig. 2a) a quantification of neuronal stem cells, i.e. Radial glia-like cells (RGL), was conducted at 1 dpi. Neither absolute cell numbers in young or older Klotho deficient mice (Fig. 2b) nor the proportion of BrdU^+^ cells (~ 5%) that were active neuronal stem cells (Fig. 2c) were statistically significantly different. Transient amplifying progenitor cells (TAPs) were also unaltered by Klotho deficiency in both absolute numbers (Fig. 2d) and relative proportion (~ 60% of cells at 1 dpi; Fig. 2e). The only significant change was a strong yet expected age-dependent reduction of stem cells (Fig. 2b) and TAPs (Fig. 2d) in both genotypes. Klotho has been proposed to be a regulator of stem cell senescence^34^. However, our data suggest that neither neuronal stem cells nor proliferation of progenitor cells in the DG are influenced by local DG-Klotho.

Neuronal stem cells also give rise to astrocytes and we therefore investigated whether DG-Klotho would affect adult gliogenesis in the DG. For this purpose, we co-stained BrdU with S100B, a specific marker of astrocytes (Fig. 2f). Roughly 3–5% of the BrdU^+^ cells at 1 dpi represent new born glia and we detected no significant changes in the total number of adult-born glia nor in the relative number of glia cells (Fig. 2g). In the hilus, sparse amounts of BrdU^+^ cells could be detected and co-staining identified them as mostly newborn glia. No differences in the absolute or relative numbers of adult-born glia were observed either. Thus, Klotho is not involved gliogenesis in the DG.

### DG-specific Klotho deficiency impacts survival of adult-born neurons in the immature state

**Fig. 3:**
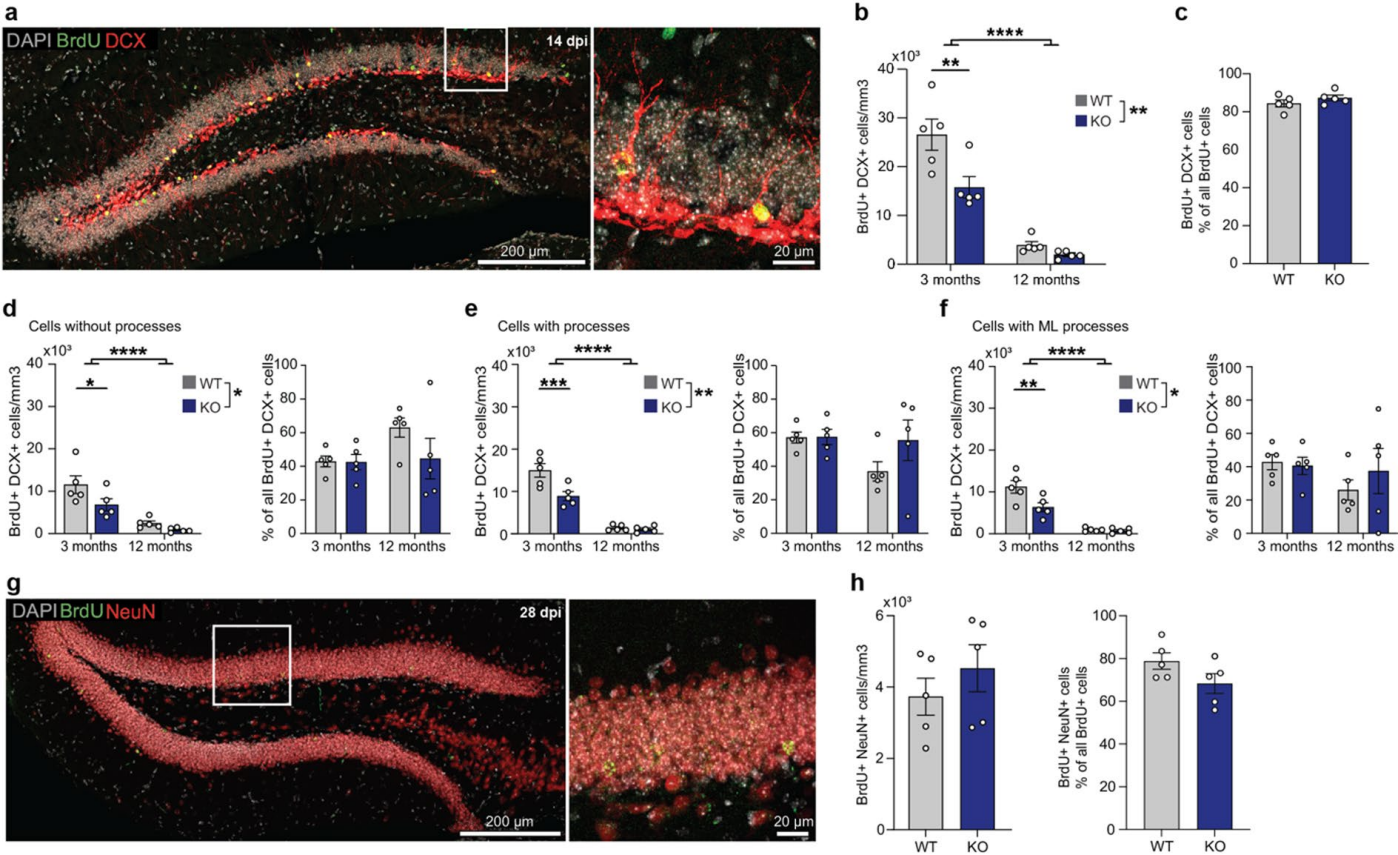
DG-specific Klotho results in a selective, temporally restricted reduction of immature neurons. (**a**) Coronal section of the DG from a 3-month-old Klotho WT mouse with DCX (doublecortin) as marker for neuronal immaturity (left). Exemplary immature neuron shown in 63x magnification (right). (**b**) Quantification of immature neurons (BrdU^+^, DCX^+^) in mice at 14 dpi in young and aged mice shows a reduction in 3 months-old-mice (*p* = 0.0013), a genotype effect (*p* = 0.0052) and an age effect (*p* < 0.0001). (**c**) Proportion of DCX^+^/BrdU^+^ immature neurons among all BrdU^+^ cells in 3-month-old mice at 14 dpi. (**d-f**) Absolute and relative quantification of immature neurons based on the presence of neuronal processes: (**d**) immature neurons without processes show a reduction in 3 months-old-mice (*p* = 0.0201), a genotype effect (*p* = 0.0262) and an age effect (*p* < 0.0001), (**e**) immature neurons with processes show a reduction in 3 months-old-mice (*p* = 0.0005), a genotype effect (*p* = 0.005) and an age effect (*p* < 0.0001), (**f**) immature neurons with processes reaching the molecular layer show a reduction in 3 months-old-mice (*p* = 0.0023), a genotype effect (*p* = 0.0161) and an age effect (*p* < 0.0001). (**g**) Coronal section of the DG from 3-month-old WT mice showing NeuN as marker for mature neurons at 28 dpi (left), with one exemplary mature adult-born neuron shown (right). (**h**) Absolute and relative quantification of adult-born mature neurons (BrdU^+^, NeuN^+^) at 28 dpi in 3-month-old mice. Error bars indicate s.e.m., either two-way ANOVA (b, d, e, f) or two-tailed students t-test (c, h) were performed, * *p* < 0.05, ** *p* < 0.01, *** < 0.001, **** *p* < 0.0001. Images (a, g) were obtained using a Leica SP8 confocal microscope and analyzed using Fiji (ImageJ 2.9.0).

To further validate and specify the reduction of BrdU^+^ cells at 7–14 dpi, co-staining with doublecortin (DCX) as marker for immature neurons was performed (Fig. 3a). At 14 dpi, we observed a genotype-dependent reduction in addition to the expected age-dependent reduction (Fig. 3b). This reduction was not accompanied with changes in the relative numbers, i.e. the proportion of DCX^+^/BrdU^+^ (immature) cells among all BrdU^+^ (newly born) cells, indicating that the deficit reflects a reduction in survival at this stage rather than a delay in maturation (Fig. 3c). We characterized these immature neurons based on morphology and differentiated them as early immature neurons without developed processes (Fig. 3d), immature neurons with processes (Fig. 3e) and late immature neurons with processes already extending to the molecular layer (ML) representing the most mature subpopulation (Fig. 3f). Klotho-deficiency resulted in a proportional reduction of immature neurons across all three subpopulations, without effect on relative numbers indicating that no accelerated or delayed progression in the immature neuron state has occurred. To determine whether the transient reduction in immature neurons at 14 dpi persisted at later stages, we examined neurons at 28 days of age using NeuN as marker for mature neurons (Fig. 3g). At 28 dpi both absolute and relative numbers of BrdU^+^/NeuN^+^ neurons were comparable between genotypes (Fig. 3h), confirming that the reduction observed during the immature phase does not persist at later stages. The proportional reduction across all immature stages, without shifts in relative distribution, together with normalization at 28 dpi, indicates that equalization of cell numbers occurs during the late maturation window (days 18–28) rather than by accelerated progression through earlier developmental stages.

## Discussion

Previous studies addressing whether brain Klotho affects AHN have been limited by the absence of conditional mouse models that allow an investigation of the consequences of loss-of-function in a cell-type specific manner. To close this gap, we generated transgenic mice with a selective deletion of the Klotho gene in granule cells of the DG (POMC-Cre; Klotho^fl/fl^ mice).

POMC is expressed transiently in dentate granule cells during early postnatal development and re-emerges during AHN. Adult-born neurons reach peak POMC expression at ~ 10–11 days after cell birth, with minimal expression before day 3^35,36^. Endogenous Klotho follows a similar pattern, being absent during the first few days of the post-mitotic stage^7^. POMC-Cre-mediated recombination occurs during the second week of neuronal age, both in postnatally generated granule neurons (when the mice are approximately two weeks old) and in adult-born granule neurons (approximately 10–11 days post-mitosis)^35,36^, deleting Klotho expression as neurons progress beyond this stage. YFP reporter staining confirmed that most dentate granule cells underwent recombination, while YFP-negative cells in the inner and outer granule cell layers may represent granule cells that escaped recombination, as well as the comparatively small populations of GABAergic interneurons or astrocytes.

Although BrdU can exert toxic effects at higher doses, the dosing regimen used here (50 mg/kg every 8 h for 3 days) falls within the range commonly employed in adult hippocampal neurogenesis studies, and similar repeated low-dose pulse-labeling paradigms are well established in the field^37,38^. Previous studies using comparable or higher cumulative dosing paradigms did not report adverse effects on proliferation or survival of adult-born DG neurons^38–40^. As WT and KO mice were subjected to the identical labeling protocol, any residual nonspecific effects of BrdU would be expected to affect both groups equally.

Using these mice, we found that Klotho expression restricted to the DG reduced AHN during days 7–17 post-mitosis. DG-specific deletion did not affect the number of stem cells, progenitor proliferation, or gliogenesis. Despite the reduction in BrdU⁺ immature neurons during this period, the number of adult-born neurons normalized by 3–4 weeks to levels that were indistinguishable from WT mice.

This transient deficit following local gene deletion is different from an organismal Klotho gene knockout that produces sustained changes across all maturation stages by regulating the neurogenic niche, stem cell quiescence, and proliferation rates^7,41^. We assume that normal proliferation is mainly driven by systemic mechanisms and that in DG specific Klotho-deficient mice surviving neurons integrate at normal levels consistent with a “circuit integration capacity” model wherein the DG maintains stable integrated neuron pools through regulated survival^42,43^. During the 2-4-week window, newly generated neurons compete for afferent inputs, output synaptic space on CA3 pyramidal cells, and trophic support^42–46^. Enhanced early attrition in Klotho-deficient cohorts reduces this competition. Surviving neurons encounter a “less crowded” environment during synapse formation and activity-dependent selection, enhancing their probability of receiving sufficient support to trigger survival signals. This interpretation is consistent with the competitive, NMDAR-mediated survival selection that normally operates during this window, in which approximately half of the generated granule cells are lost through cell-autonomous but input-dependent mechanisms^42,47,48^. Candidate molecular mechanisms for the local Klotho-dependent survival deficit include Klotho’s regulation of Ca²⁺ homeostasis and oxidative stress responses^49^ and its attenuation of BDNF-Ca²⁺ signaling^50^.

In conclusion, DG-expressed Klotho is required for AHN during a temporally restricted window (days 7–17 post-mitosis). Despite enhanced attrition, mature neuron numbers normalize by 3–4 weeks, presumably through reduced competition for integration slots and trophic support. Local DG-Klotho impacts early post-mitotic maturation, while proliferation and mature neuron numbers can be maintained through other Klotho sources. These findings complement systemic studies and provide a foundation for dissecting anatomically and temporally distinct contributions of Klotho to AHN.

## Data Availability

Further details or any additional information required to reanalyze the data reported in this paper are available from the lead contact (anja.oelschlegel@lin-magdeburg.de) upon request.

## References

[1] Kuro-o, M. et al. Mutation of the mouse klotho gene leads to a syndrome resembling ageing. *Nature* **390**, 45–51 (1997). 9363890 10.1038/36285

[2] Duce, J. A. et al. Gene profile analysis implicates Klotho as an important contributor to aging changes in brain white matter of the rhesus monkey. *Glia* **56**, 106–117 (2008). 17963266 10.1002/glia.20593

[3] Massó, A. et al. Secreted and Transmembrane αKlotho Isoforms Have Different Spatio-Temporal Profiles in the Brain during Aging and Alzheimer’s Disease Progression. *PLoS ONE*. **10**, e0143623 (2015). 26599613 10.1371/journal.pone.0143623 PMC4658185

[4] Nagai, T. et al. Cognition impairment in the genetic model of aging klotho gene mutant mice: a role of oxidative stress. *FASEB J.* **17**, 50–52 (2003). 12475907 10.1096/fj.02-0448fje

[5] Dubal, D. B. et al. Life extension factor klotho enhances cognition. *Cell. Rep.* **7**, 1065 (2014). 24813892 10.1016/j.celrep.2014.03.076 PMC4176932

[6] Shardell, M. et al. Plasma Klotho and Cognitive Decline in Older Adults: Findings From the InCHIANTI Study. *GERONA* **71**, 677–682 (2016). 10.1093/gerona/glv140 PMC5007737 26297657

[7] Laszczyk, A. M. et al. Klotho regulates postnatal neurogenesis and protects against age-related spatial memory loss. *Neurobiol. Aging*. **59**, 41–54 (2017). 28837861 10.1016/j.neurobiolaging.2017.07.008 PMC5612914

[8] Kurosu, H. et al. Suppression of Aging in Mice by the Hormone Klotho. *Science* **309**, 1829–1833 (2005). 16123266 10.1126/science.1112766 PMC2536606

[9] Dubal, D. B. et al. Life extension factor klotho prevents mortality and enhances cognition in hAPP transgenic mice. *J. Neurosci.* **35**, 2358–2371 (2015). 25673831 10.1523/JNEUROSCI.5791-12.2015 PMC4323521

[10] Leon, J. et al. Peripheral Elevation of a Klotho Fragment Enhances Brain Function and Resilience in Young, Aging, and α-Synuclein Transgenic Mice. *Cell. Rep.* **20**, 1360–1371 (2017). 28793260 10.1016/j.celrep.2017.07.024 PMC5816951

[11] Hu, M. C. et al. Renal Production, Uptake, and Handling of Circulating αKlotho. *JASN* **27**, 79–90 (2016). 25977312 10.1681/ASN.2014101030 PMC4696570

[12] Park, C. et al. Platelet factors are induced by longevity factor klotho and enhance cognition in young and aging mice. *Nat. Aging*. **3**, 1067–1078 (2023). 37587231 10.1038/s43587-023-00468-0 PMC10501899

[13] Prud’homme, G. J., Kurt, M. & Wang, Q. Pathobiology of the Klotho Antiaging Protein and Therapeutic Considerations. *Front. Aging*. **3**, 931331 (2022). 35903083 10.3389/fragi.2022.931331 PMC9314780

[14] Clinton, S. M. et al. Expression of klotho mRNA and protein in rat brain parenchyma from early postnatal development into adulthood. *Brain Res.* **1527**, 1–14 (2013). 23838326 10.1016/j.brainres.2013.06.044 PMC3756829

[15] Salech, F. et al. Local Klotho Enhances Neuronal Progenitor Proliferation in the Adult Hippocampus. *J. Gerontol. Biol. Sci. Med. Sci.* **74**, 1043–1051 (2019). 10.1093/gerona/glx248 29300914

[16] Dias, G. P. et al. Intermittent fasting enhances long-term memory consolidation, adult hippocampal neurogenesis, and expression of longevity gene Klotho. *Mol. Psychiatry*. **26**, 6365–6379 (2021). 34031536 10.1038/s41380-021-01102-4 PMC8760057

[17] Ho, W. Y., Navakkode, S., Liu, F., Soong, T. W. & Ling, S. C. Deregulated expression of a longevity gene, Klotho, in the C9orf72 deletion mice with impaired synaptic plasticity and adult hippocampal neurogenesis. *acta neuropathol. commun.* **8**, 155 (2020). 32887666 10.1186/s40478-020-01030-4 PMC7473815

[18] Shors, T. J. et al. Neurogenesis in the adult is involved in the formation of trace memories. *Nature* **410**, 372–376 (2001). 11268214 10.1038/35066584

[19] Clelland, C. D. et al. A Functional Role for Adult Hippocampal Neurogenesis in Spatial Pattern Separation. *Science* **325**, 210-213 (2009). 10.1126/science.1173215 PMC2997634 19590004

[20] Jessberger, S. et al. Dentate gyrus-specific knockdown of adult neurogenesis impairs spatial and object recognition memory in adult rats. *Learn. Mem.* **16**, 147–154 (2009). 19181621 10.1101/lm.1172609 PMC2661246

[21] Sahay, A. et al. Increasing adult hippocampal neurogenesis is sufficient to improve pattern separation. *Nature* **472**, 466-470 (2011). 10.1038/nature09817 PMC3084370 21460835

[22] Sahay, A., Wilson, D. A. & Hen, R. Pattern Separation: A Common Function for New Neurons in Hippocampus and Olfactory Bulb. *Neuron* **70**, 582–588 (2011). 21609817 10.1016/j.neuron.2011.05.012 PMC3109085

[23] Frechou, M. A. et al. Adult neurogenesis improves spatial information encoding in the mouse hippocampus. *Nat. Commun.* **15**, 6410 (2024). 39080283 10.1038/s41467-024-50699-x PMC11289285

[24] Kuhn, H., Dickinson-Anson, H. & Gage, F. Neurogenesis in the dentate gyrus of the adult rat: age-related decrease of neuronal progenitor proliferation. *J. Neurosci.* **16**, 2027–2033 (1996). 8604047 10.1523/JNEUROSCI.16-06-02027.1996 PMC6578509

[25] Ben Abdallah, N. M. B., Slomianka, L., Vyssotski, A. L. & Lipp, H. P. Early age-related changes in adult hippocampal neurogenesis in C57 mice. *Neurobiol. Aging*. **31**, 151–161 (2010). 18455269 10.1016/j.neurobiolaging.2008.03.002

[26] Aimone, J. B. et al. Regulation and Function of Adult Neurogenesis: From Genes to Cognition. *Physiol. Rev.* **94**, 991–1026 (2014). 25287858 10.1152/physrev.00004.2014 PMC4280160

[27] Kase, Y., Shimazaki, T. & Okano, H. Current understanding of adult neurogenesis in the mammalian brain: how does adult neurogenesis decrease with age? *Inflamm. Regen*. **40**, 10 (2020). 32566044 10.1186/s41232-020-00122-x PMC7302355

[28] Culig, L., Chu, X. & Bohr, V. A. Neurogenesis in aging and age-related neurodegenerative diseases. *Ageing Res. Rev.* **78**, 101636 (2022). 35490966 10.1016/j.arr.2022.101636 PMC9168971

[29] Amelchenko, E. M. et al. Age-related decline in cognitive flexibility is associated with the levels of hippocampal neurogenesis. *Front. Neurosci.* **17**, 1232670 (2023). 37645372 10.3389/fnins.2023.1232670 PMC10461065

[30] Mchugh, T. et al. Dentate Gyrus NMDA Receptors Mediate Rapid Pattern Separation in the Hippocampal Network. *Sci. (New York N Y)*. **317**, 94–99 (2007). 10.1126/science.1140263 17556551

[31] Fanaei-Kahrani, Z. et al. Distinct effects of aging and klotho deletion on the choroid plexus. *GeroScience* (2026). 10.1007/s11357-026-02196-w 10.1007/s11357-026-02196-w PMC13356169 41879938

[32] Bankhead, P. et al. QuPath: Open source software for digital pathology image analysis. *Sci. Rep.* **7**, 16878 (2017). 29203879 10.1038/s41598-017-17204-5 PMC5715110

[33] Boulanger, J. J. A simple histological technique to improve immunostaining when using DNA denaturation for BrdU labelling. *Journal Neurosci. Methods* **259**, 40-46 (2016). 10.1016/j.jneumeth.2015.11.006 26620201

[34] Bian, A., Neyra, J. A., Zhan, M. & Hu, M. C. Klotho, stem cells, and aging. *Clin. Interv Aging*. **10**, 1233–1243 (2015). 26346243 10.2147/CIA.S84978 PMC4531025

[35] Overstreet, L. S. et al. A Transgenic Marker for Newly Born Granule Cells in Dentate Gyrus. *J. Neurosci.* **24**, 3251–3259 (2004). 15056704 10.1523/JNEUROSCI.5173-03.2004 PMC6730035

[36] Gao, X., Arlotta, P., Macklis, J. D. & Chen, J. Conditional Knock-Out of β- *Catenin* in Postnatal-Born Dentate Gyrus Granule Neurons Results in Dendritic Malformation. *J. Neurosci.* **27**, 14317–14325 (2007). 18160639 10.1523/JNEUROSCI.3206-07.2007 PMC6673436

[37] Nowakowski, R. S., Lewin, S. B. & Miller, M. W. Bromodeoxyuridine immunohistochemical determination of the lengths of the cell cycle and the DNA-synthetic phase for an anatomically defined population. *J. Neurocytol*. **18**, 311–318 (1989). 2746304 10.1007/BF01190834

[38] Taupin, P. BrdU immunohistochemistry for studying adult neurogenesis: paradigms, pitfalls, limitations, and validation. *Brain Res. Rev.* **53**, 198–214 (2007). 17020783 10.1016/j.brainresrev.2006.08.002

[39] Cameron, H. A. & Mckay, R. D. G. Adult neurogenesis produces a large pool of new granule cells in the dentate gyrus. *J. Comp. Neurol.* **435**, 406–417 (2001). 11406822 10.1002/cne.1040

[40] Hancock, A., Priester, C., Kidder, E. & Keith, J. R. Does 5-bromo-2′-deoxyuridine (BrdU) disrupt cell proliferation and neuronal maturation in the adult rat hippocampus in vivo? *Behav. Brain. Res.* **199**, 218–221 (2009). 19121338 10.1016/j.bbr.2008.11.050 PMC4154233

[41] Kuro-o, M. The Klotho proteins in health and disease. *Nat. Rev. Nephrol.* **15**, 27–44 (2019). 30455427 10.1038/s41581-018-0078-3

[42] Tashiro, A., Sandler, V. M., Toni, N., Zhao, C. & Gage, F. H. NMDA-receptor-mediated, cell-specific integration of new neurons in adult dentate gyrus. *Nature* **442**, 929–933 (2006). 16906136 10.1038/nature05028

[43] Toni, N. et al. Neurons born in the adult dentate gyrus form functional synapses with target cells. *Nat. Neurosci.* **11**, 901–907 (2008). 18622400 10.1038/nn.2156 PMC2572641

[44] Bergami, M. et al. Deletion of TrkB in adult progenitors alters newborn neuron integration into hippocampal circuits and increases anxiety-like behavior. *Proc. Natl. Acad. Sci. U S A*. **105**, 15570–15575 (2008). 18832146 10.1073/pnas.0803702105 PMC2557028

[45] McAvoy, K. M. et al. Modulating Neuronal Competition Dynamics in the Dentate Gyrus to Rejuvenate Aging Memory Circuits. *Neuron* **91**, 1356–1373 (2016). 27593178 10.1016/j.neuron.2016.08.009 PMC5033725

[46] Adlaf, E. W. et al. Adult-born neurons modify excitatory synaptic transmission to existing neurons. *eLife* **6**, e19886 (2017). 28135190 10.7554/eLife.19886 PMC5279947

[47] Kempermann, G., Jessberger, S., Steiner, B. & Kronenberg, G. Milestones of neuronal development in the adult hippocampus. *Trends Neurosci.* **27**, 447–452 (2004). 15271491 10.1016/j.tins.2004.05.013

[48] Dayer, A. G., Ford, A. A., Cleaver, K. M., Yassaee, M. & Cameron, H. A. Short-term and long‐term survival of new neurons in the rat dentate gyrus. *J. Comp. Neurol.* **460**, 563–572 (2003). 12717714 10.1002/cne.10675

[49] Zeldich, E. et al. The Neuroprotective Effect of Klotho is Mediated via Regulation of Members of the Redox System. *J. Biol. Chem.* **289**, 24700–24715 (2014). 25037225 10.1074/jbc.M114.567321 PMC4148892

[50] Cararo-Lopes, M. M. et al. Overexpression of α-Klotho isoforms promotes distinct Effects on BDNF-Induced Alterations in Dendritic Morphology. *Mol. Neurobiol.* **61**, 9155–9170 (2024). 38589756 10.1007/s12035-024-04171-y PMC11496329

